# Predictable transcranial magnetic stimulation suppresses corticospinal excitability: a TMS experiment

**DOI:** 10.1007/s00221-025-07091-y

**Published:** 2025-05-04

**Authors:** Napat Sriutaisuk, Elizabeth A. Franz

**Affiliations:** 1 Action Brain and Cognition Lab, Department of Psychology, University of Otago, Dunedin, New Zealand; 2 William James Building, 4th Floor 412, Psychology, 275 Leith Walk, Dunedin, 9016 New Zealand

**Keywords:** Motor skill learning, Corticospinal excitability (CSE), Transcranial magnetic stimulation (TMS), TMS predictability, Electromyography (EMG), First dorsal interosseous (FDI)

## Abstract

Motor skill learning plays a crucial role in human functioning and is often studied using transcranial magnetic stimulation (TMS) to assess corticospinal excitability (CSE). CSE, which reflects the motor system’s responsiveness, is compared across experimental conditions to determine whether a factor facilitates or inhibits motor skill learning. However, variability in stimulation predictability may confound interpretation of CSE measures. In this study, we examined the impact of TMS predictability on CSE by comparing motor-evoked potential (MEP) responses under predictable and unpredictable conditions. Twenty right-handed participants underwent TMS-EMG recordings while observing either a predictable visual cue (a moving white bar) or an unpredictable visual cue (a static white bar). MEP amplitudes were recorded from the first dorsal interosseous (FDI) muscle and normalized across participants. Results showed that unpredictable TMS produced significantly larger MEP amplitudes compared to predictable stimulation. The findings suggest that the predictability of TMS substantially modulates CSE, potentially confounding the results in previous TMS studies on action observation and motor facilitation. Ensuring consistent stimulation predictability across experimental conditions is therefore essential for accurately interpreting TMS-induced CSE changes.

**Supplementary Information:**

The online version contains supplementary material available at 10.1007/s00221-025-07091-y.

Motor skill learning relies on the dynamic interplay of neural excitability and inhibition (Naish et al. 2014), often studied through transcranial magnetic stimulation (TMS). TMS-induced motor-evoked potentials (MEPs) provide a measure of corticospinal excitability (CSE), offering insights into neuroplasticity mechanisms (Pascual-Leone et al. 2005; Kleim and Jones 2008).

Action Observation (AO) has been shown to reliably increase MEP amplitudes, suggesting CSE facilitation (e.g. Fadiga et al. 1995; Gangitano et al. 2001; Loporto et al. 2012; 2013; Meers et al. 2020). However, experimental conditions tend to vary in predictability, introducing a confound.

While some studies attempted to reduce predictability—such as by randomly delivering TMS at two possible time points (Loporto et al. 2012; Riach et al. 2018)—these designs still allowed partial predictability. Other studies (e.g., Takei et al. 2005; Tran et al. 2021) have also tried to isolate the effect of predictability by using internal timing or structured visual cues, but their methods introduced potential confounds that complicate interpretation. To address these issues, we used a visual bar that triggered stimulation when full, providing a clear and externally cued indication of timing across conditions.

While previous research suggests that AO facilitates CSE, the predictability of TMS still varies across conditions, making it difficult to isolate its precise effects. Furthermore, AO studies showed conditions with unpredictable TMS resulted in a smaller MEP amplitude than predictable TMS. However, studies that directly investigate TMS predictability found that unpredictable TMS increases MEP amplitude compared to predictable TMS. The current study aimed to investigate the influence of TMS predictability on CSE, expanding upon the framework established by Tran et al. (2021). We hypothesised that predictable TMS would result in a lower MEP amplitude than unpredictable stimulation.

## Methods

### Participants

Participants were recruited through university posters and social media. Based on prior studies, the sample size was determined assuming α = 0.05 and power = 0.80 (Takei et al. 2005; Tran et al. 2021).

All participants had normal or corrected-to-normal eyesight. Participants were excluded if their handedness scores indicated non-right-handedness (< 50 on the EHI questionnaire; Oldfield 1971) or reported any history of seizure/epilepsy, stroke, severe head injury, fainting, metal in their head, welding, injury in their eye(s) by a metal, implanted devices (e.g., pacemaker), frequent headache, or any chance of pregnancy to prevent potential health risks (Keel et al. 2001). They were asked to refrain from consuming caffeine for at least two hours before the experiment and from engaging in strenuous activities for at least 24 h before the experiment began. Ethical approval was obtained from University of Otago Human Ethics Committee, approval number H22/059 and funded in part by the Marsden Fund of the Royal Society of New Zealand. All participants provided written informed consent before participating. Participant demographics information is summarised in Table 1.

**Table 1:** Descriptive characteristics of the participants

Variable	Value
N (right-handed)	20 participants
Age (M ± SD)	23.1 ± 4.16 years
Gender	5 male, 15 female
EHI score (M ± SD)	79 ± 3.30
rMT (M ± SD)	52.6% ± 2.25% MSO

## Materials and recording protocol

### Questionnaires

Handedness was assessed using the 10-item EHI (Oldfield 1971). TMS safety screening was conducted using the 14-item TASS (Keel et al. 2001). Participants with contraindications (e.g., epilepsy, metal implants, pregnancy) were excluded.

### Electromyography recording

The electromyographic (EMG) activity of the bilateral First Dorsal Interosseous (FDI) was recorded using Kendall™ 200 series foam electrodes (Cardinal Health, Dublin, OH, USA) positioned 2 cm apart around the midpoint of the FDI muscle. A ground electrode was placed on the ulnar styloid process to reference each hand and reduce interference (Lee and Kruse 2008; Riach et al. 2018). EMG signals were recorded using BIO Amps, which applied a hardware band-pass filter from 10 to 5000 Hz and sampled at 10 kHz. This preprocessing is standard for EMG acquisition and attenuates low-frequency drift and high-frequency noise before digitisation. The signals were then transmitted to the PowerLab/4SP, which was connected to a computer with LabChart 7.

### Transcranial magnetic stimulation and neuronavigation

A single-pulse monophasic TMS was delivered using a Magstim 200^2^ stimulator connected to a figure-of-eight coil (two 70 mm loops). The coil was placed over the left motor cortex hotspot for the right FDI muscle, approximately 4 cm lateral and 1.5 cm anterior to Cz (Loporto et al. 2013), tangential to the scalp with the handle angled 45° posterior–anterior.

Neuronavigation was used to guide coil positioning using the NDI Vega ST system and InVesalius 3.1.99998 software (Souza et al. 2018). Participants wore a headband with reflective markers for motion tracking.

To identify the optimal scalp position (OSP), suprathreshold stimulations were delivered in a 1 × 1 cm grid around the motor hotspot using an estimated rMT + 5% intensity. The site producing the largest MEP was marked on InVesalius and used for formal rMT estimation using the MTAT 2.0 tool.

The resting motor threshold (rMT) was estimated using the MTAT 2.0 tool (Awiszus and Borckardt 2011) and confirmed by identifying the lowest stimulation intensity that reliably elicited ≥ 5 out of 10 MEPs ≥ 0.05 mV on the OSP. TMS pulses were delivered at 120% of rMT during the experiment, consistent with prior studies (e.g., Perera et al. 2024; Reid and Serrien 2014).

### Experimental procedure

Participants completed the EHI and TASS questionnaires before scheduling. On the experiment day, participants were seated roughly 110 cm from the screen and fitted with a headband containing a rigid body for neuronavigation (Souza et al. 2018). After cleaning the skin with isopropyl alcohol wipes, three EMG electrodes were attached to each hand to record MEPs. The OSP and rMT were identified in the left hemisphere. Participants were instructed to remain still and focus on the centre of the screen throughout the session (Wright et al. 2018).

**Fig. 1:**
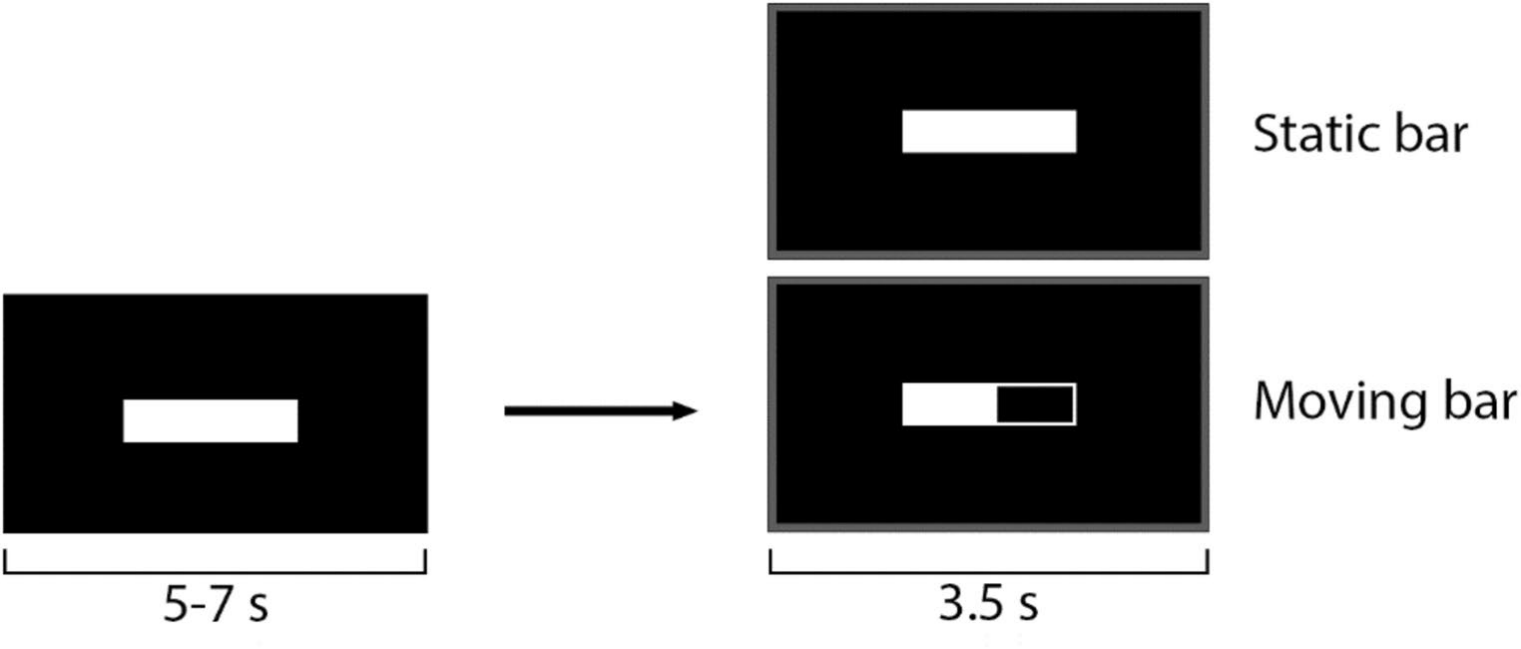
Experimental procedure for predictable (moving bar) and unpredictable (static bar) TMS. The static white bar lasts for 5 to 7 s before the program pseudo-randomly chooses between a predictable moving bar or an unpredictable static bar condition, each lasting 3.5 s

The independent variable was the TMS predictability (predictable and unpredictable). Each condition was presented using E-prime 3.0 (Psychology Software Tools, Inc. 2016) on a 24-inch LCD screen (1920 × 1080, 60 Hz). Figure 1 shows the experimental procedure used to present the experimental conditions.

Each trial began with a static white bar displayed for a random interval (5–7 s). The program then randomly selected a predictable or unpredictable condition. In the predictable condition, the bar emptied and refilled gradually over 3.5 s, with a TMS pulse delivered at full. In the unpredictable condition, the static bar remained unchanged for 3.5 s before TMS delivery, preventing temporal prediction.

Each condition was presented 20 times using identical visual stimuli devoid of human/animal content to isolate predictability (Fu and Franz 2014; Rizzolatti and Craighero 2005).

### Data analysis

EMG data were recorded using LabChart 7 (ADInstruments 2023) at a 10 kHz sampling rate, band-pass filtered online (10–5000 Hz), and continuously monitored throughout the experiment to detect spontaneous muscle activity. Trials were segmented offline into 9-second windows (–3.5 to + 5.5 s) and down-sampled to 2 kHz for analysis.

Analysis was conducted using R (v4.4.1; R Core Team 2021). All 20 participants completed the study. No dropout occurred, but 21 trials (2.63%) were excluded due to excessive pre-stimulus EMG noise (± 2.5 SD filter; Loporto et al. 2012; Riach et al. 2018; Wright et al. 2018).

Pre-TMS EMG was assessed in two windows: − 3000 to − 1500 ms (baseline tone) and − 1500 to − 5 ms (anticipatory tension). EMG was quantified as the voltage range (maximum – minimum) per window, and mean values were calculated for each participant per condition. Normality and variance assumptions were assessed using the Shapiro-Wilk and Levene’s tests, and group comparisons were conducted using either a two-sample t-test or Wilcoxon rank-sum test, depending on assumption outcomes.

Peak-to-peak MEP amplitudes were calculated from the high-pass filtered signal (20 Hz, zero-phase Butterworth) on a single-trial basis, then averaged by condition (predictable, unpredictable) for each participant. The primary outcome measure was the raw MEP amplitude from the contralateral FDI muscle. Z-scored MEP amplitudes were also computed to assess the robustness of the effect (Meers et al. 2020; Riach et al. 2018; Vallence et al. 2023). Normality was confirmed with Shapiro-Wilk tests, and paired t-tests were applied to both raw and z-scored data.

Waveforms were grand-averaged and time-locked to the TMS pulse (0 ms) to visualise response timing. These are presented for illustrative purposes only and were not used for statistical analysis.

Finally, linear regression analysis examined the influence of predictability, stimulation intensity, age, gender, and handedness on MEP amplitudes. Residual diagnostics were performed to check for outliers and violations of regression model assumptions. Detailed scripts and steps are provided in Supplementary Material 1.

## Result

Pre-TMS EMG was analysed to confirm participant relaxation. The − 1500 to − 5 ms window met normality and variance assumptions; a two-sample t-test showed no significant difference between conditions (t [37.67] = 0.61, *p* =.543). The − 3000 to − 1500 ms window violated normality; a Wilcoxon rank-sum test indicated no significant difference between conditions (W = 221, *p* =.583).

**Fig. 2:**
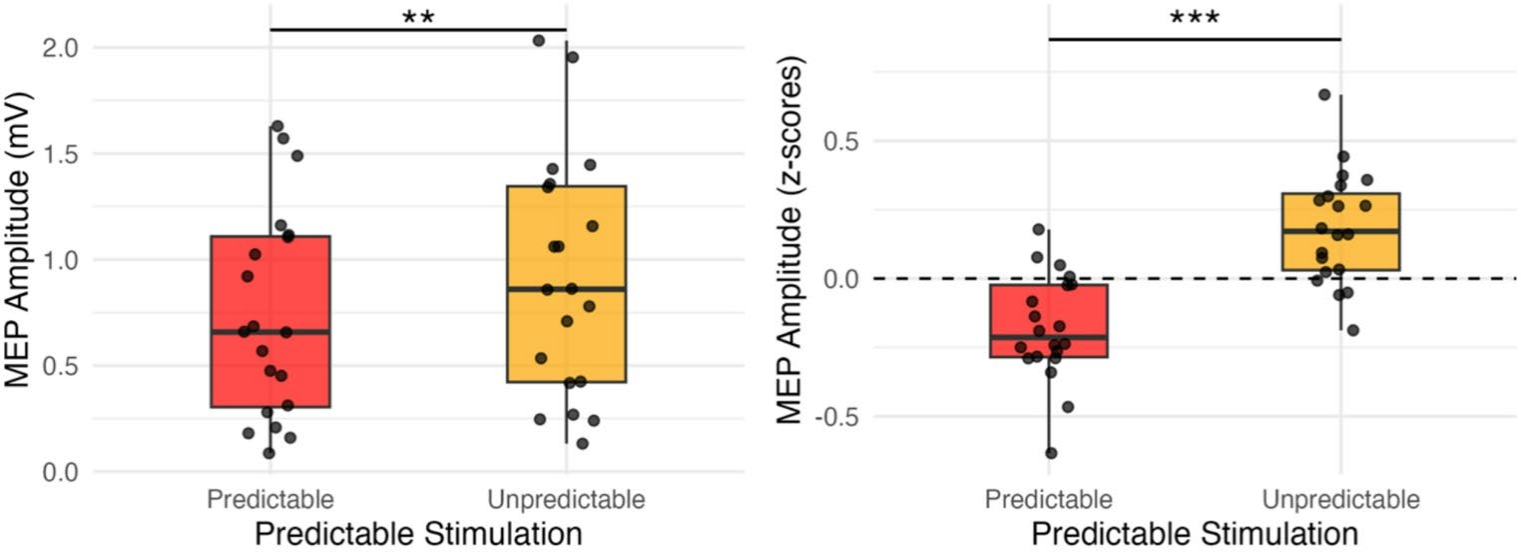
Boxplot showing MEP amplitudes (raw [left] and z-scores [right]) for predictable and unpredictable stimulation conditions. Individual participant values are displayed as black jittered dots. **p <.005, ***p <.001

**Fig. 3:**
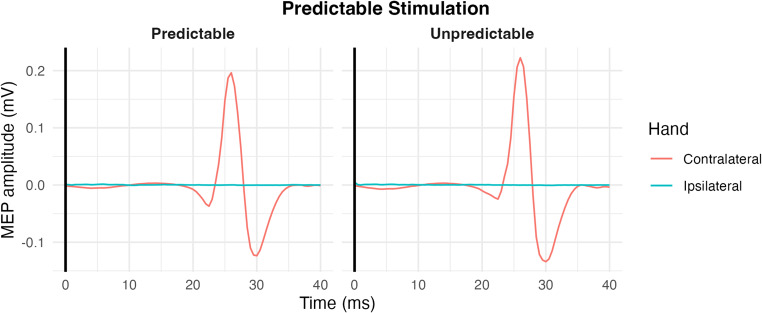
Grand-averaged MEP waveforms (0–40 ms post-TMS) from contralateral (red) and ipsilateral (blue) FDI muscles in predictable and unpredictable conditions. Each trace represents the average across participants. These waveforms are shown for visualisation only. MEP amplitudes appear lower than reported values due to inter-trial latency variability and phase cancellation

As shown in Fig. 2, the MEP amplitudes were larger under unpredictable (0.906 ± 0.12 mV) than predictable (0.743 ± 0.11 mV) conditions. A Shapiro-Wilk test on the difference scores confirmed the normality assumption (W = 0.93, *p* =.16). A paired t-test on the MEP amplitude showed a significant difference (t [19] = 3.69, *p* =.0015) with a large effect size (d = 0.83, 95% CI [0.31, 1.33]). This effect was also evident in z-scored amplitudes (t [19] = 4.15, *p* <.001, d = 0.93, 95% CI [0.39, 1.45]), supporting a robust suppression in corticospinal excitability under predictable stimulation. Figure 3 shows the averaged MEP waveforms, highlighting the reduced peak-to-peak amplitude in the predictable condition compared to the unpredictable one.

A linear regression using raw MEP amplitudes was significant (F [6, 33] = 3.72, *p* =.006; Adjusted R² = 29.52%), but predictability was not a significant predictor (β = − 0.178, *p* =.21). Instead, stimulation intensity and handedness—both stable within subjects but highly variable between participants—were significant predictors (both *p* <.005). To account for this between-subject variability, a second model using z-scored amplitudes was run, which improved model fit (F [6, 33] = 4.98, *p* =.001; Adjusted R² = 37.98%) and identified predictability as the only significant predictor (β = − 0.366, *p* <.001). Residuals showed no violations of model assumptions.

## Discussion

This experiment aimed to determine the potential influence of the predictability of the TMS on the MEP amplitude. The result supported the hypothesis that a predictable TMS would result in a lower MEP amplitude than unpredictable stimulation. Other variables that potentially could confound the result, such as the stimulation intensity, age, gender, and handedness, did not significantly affect CSE.

This result further highlighted that predictability suppresses CSE facilitation, which may have confounded results in earlier studies. Despite the difference in TMS predictability, AO has been consistently shown to increase MEP amplitude relative to baseline. These effects were often attributed to mirror neuron activation and observational learning (e.g. Fadiga et al. 1995 or Meers et al. 2020).

The current results suggest that previously reported AO-induced CSE facilitation may, in part, reflect predictability effects rather than AO per se. While this result does not negate the role of AO in CSE facilitation, it emphasises the importance of controlling the stimulation predictability in future studies.

The present study addressed methodological limitations in prior work by Takei et al. (2005) and Tran et al. (2021), who attempted to isolate predictability effects but introduced key design confounds. Takei et al. relied on internal estimation of timing, while Tran et al. used a rotating clock paradigm where most trials were predictable. These methods may have elicited attentional or expectancy-related responses (Schröger 1997; Franz and Miller 2002) and lacked neuronavigation, raising concerns about stimulation consistency. In contrast, we synchronised stimulation to a completed visual bar, ensured matched visual stimuli, used equal trial numbers across conditions, and implemented neuronavigation for accurate targeting. Despite these improvements, our findings aligned with both Takei et al. and Tran et al., showing that predictable TMS produces reduced MEP amplitudes.

While raw MEP amplitudes are commonly reported, inter-individual variability in baseline excitability can obscure condition effects in models that include covariates. Although the raw-data t-test showed a significant difference, regression revealed that stimulation intensity and handedness influenced MEPs, masking the experimental effect. Z-score normalisation mitigated this issue, revealing predictability as the only significant predictor. Thus, while we report both raw and normalised data, our interpretation is based primarily on the z-scored values due to their robustness in accounting for participant variability.

Several mechanisms can explain the suppression of MEP amplitude during predictable stimulation. One key account is predictive coding, which suggests that the brain actively generates internal models to anticipate sensory consequences of incoming events (Friston 2005). When an input matches the prediction, neural responses are attenuated to conserve processing resources (Bäß et al. 2008). Betti et al. (2023) propose that anticipated sensorimotor inputs are actively gated or inhibited to prevent redundant activation, especially when no action is required.

In contrast, unpredictable stimulation may increase MEP amplitude due to greater attentional engagement or violations of expectancy. According to Schröger’s (1997) pre-attentive activation model, deviations from anticipated sensory events elicit enhanced processing to update the internal model. This heightened state of responsiveness may lead to greater CSE, thereby increasing MEP amplitude.

The current experiment used only visual stimuli to cue predictable and unpredictable TMS. Thus, this study does not determine whether the difference in MEP amplitude occurs purely due to the TMS predictability or to the predictability of the visual information. Further research on stimulation predictability might consider using sound or other sensory cues to differentiate different forms of stimulus predictability.

Like Tran et al. (2021), our study confounds predictability with stimulus motion (a moving bar here, a clock hand in their study), so the ‘motion’ factor and the pure predictability factor remains to be disentangled. Although such non-embodied motion likely does not engage the mirror neuron system (Fu and Franz 2014), it could still influence MEP amplitude in ways that static cues might not. Thus, future studies could disentangle motion from predictability by using static stimuli that change in a temporally predictable or unpredictable fashion (e.g., a colour-changing dot).

In conclusion, this study reinforces the necessity of controlling TMS predictability in CSE research. Predictable stimulation significantly reduces MEP amplitude, suggesting that prior studies may have overestimated AO-induced CSE facilitation due to unaccounted predictability effects. Controlling this factor will allow for more precise conclusions about CSE modulation and its underlying mechanisms.

## Electronic Supplementary Material

Below is the link to the electronic supplementary material.

Supplementary Material 1

## Data Availability

Detailed data analysis scripts, including preprocessing and statistical tests, are available at the following GitHub repository: https://gapnapats.github.io/PredictableTMSStimulation/PAT_1-Analysis.html. This repository contains a knitted R markdown to present the detailed steps of the data analysis.
