# Supplementary material for: Predictable transcranial magnetic stimulation suppresses corticospinal excitability: a TMS experiment
Source: Exp Brain Res. 2025 May 4;243(6):134. doi: 10.1007/s00221-025-07091-y (PMC12050229; doi:10.1007/s00221-025-07091-y)
Supplement: Supplementary file 1 — Supplementary Material 1 [file 221_2025_7091_MOESM1_ESM.docx]

**Supplementary Materials**

**Supplementary Materials 1**

Detailed data analysis scripts, including preprocessing and statistical tests, are available at the following GitHub repository:

https://gapnapats.github.io/PredictableTMSStimulation/PAT_1-Analysis.html

This repository contains a knitted R markdown to present the detailed steps of the data analysis.
